# Duties and Responsibilities of the Dental Profession

**Published:** 1864-12

**Authors:** D. K. Boutelle

**Affiliations:** Manchester, N.H.


### DENTAL REGISTER OF THE WEST.

VOL. XVIII.] DECEMBER, 1864. [No. 12.

### Original Essays and Communications.

### DUTIES AND RESPONSIBILITIES OF THE DENTAL PROFESSION.

Read before the Merrimack Valley Dental Association, November 3d, 1864.

BY D. K. BOUTELLE.

Were we to assert it to be the duty of every man who offers himself to the public as a Dentist, to be properly qualified for that position, and then to perform all operations that come under his treatment to the best of his ability, and always to recommend just such as he sincerely thinks for the best interest of his patients, would be simply to affirm that every Dentist ought to be an honest man.

And that he is responsible for the effect or non-effect of his operations, will not, perhaps, be questioned by any.

But that the Dental Profession is responsible for the condition of the teeth of the whole community, whether the individuals composing that community, all come under our special manipulations or not; and that it is the duty of our profession, eventually, to do away, almost, if not entirely, the necessity of the Dentist at all, may not be so apparent to every mind.

To these points I propose to call the attention of this meeting for a few moments.

Is any one startled at the idea, that we, as a profession, are responsible for the teeth of that part of the community who never call on us for our advices or service, and who seem to have very little, if any, appreciation of the value of these organs, and quite as little idea of the means necessary for their preservation, let us ask him who is responsible for the education or non-education of the rising generation? Is it that generation itself or the one preceding it, which is already able, in some degree, to appreciate the benefit of education?

Who is responsible for the health of a city or community? Is it the mass of the people? those most affected by disease when it sweeps through their ranks? Or is it not rather the board of health—the government and the more educated class, those better able to understand the causes that produce, and the means that prevent disease?

It is not enough that the physician is able to cure the specific cases of a contagious or epidemic disease that come under his treatment. But if he be a true man—a true physician—is truly imbued with the divine spirit of the healing art, he bends his mind to discover the specific causes, and then, not only the means of cure but also the means of prevention. And not only will he do this, but when he thinks he has found it, he does not put his light under a bushel, or lock it up in his medicine case, nor yet alone in his own brain; but he publishes it to the world. He lets his light shine. He not only *lets* it shine but he *makes* it shine. He enforces it upon the public mind by precept upon precept until he gains attention and thereby educates the people on that special subject.

What a glorious example, in point, has the world in the case of vaccination, as a preventative of small pox; and yet, were that left to the masses, who are benefited by it, to execute, without the continuous efforts of physicians and governments and scientific men, the name of Jenner would be forgotten, and the art of vaccination become unknown, except to a few, and they would keep it to themselves. Even now, when its benefit is so fully shown by the experience of the world, whenever that terrible scourge, small pox, visits any locality, how many are surprised by its attack, not having availed themselves of the benefit of the great preventative. Now who is responsible for such neglect? We say the physician—scientific men—those who should, and must be educators.

He who by thus educating the public mind and thereby prevents whole communities from falling victims to some fell disease, although the world at large may not even know what he has done for it, has really done a greater service than he who has cured a hundred specific cases and thereby gained for himself great renown.

Again, the philanthropist in his labors of love and benevolence, is not content merely to pluck here and there an individual from the fires of intemperance, the dens of vice and pollution, or the chains of human bondage, as brands from the burning,—not yet, even to save whole classes of erring, and wronged ones,

"Who've slipped in life's too slippery paths."

or been crushed beneath the cruel heel of oppression, and place them back again in society, though scathed and scarred by the burning hells through which they have passed; but he studies to devise some means to save whole generations from the temptations, the falls, and the evils which others have experienced, and thereby prevent the very necessity of reform and of reformers. When he thinks he has discovered the means, which if used, may accomplish the end he has at heart, he does not hoard them merely to speculate on in his own mind, or with which to regale a friend and help him to kill an, otherwise, idle hour, but he goes to work. He sends out a Gough and a Hawkens to preach temperance, a Coles to preach and write and publish the abominations of tobacco; a Thompson, a Phillips, and a Douglas to thunder in the ears of the community the deep damnation of human slavery. And so of all the evils he attempts to reform. He enforces his thought with all the powers of reason and eloquence and wit he can command. He publishes tracts, books, periodicals, and newspaper articles, and sends them forth broadcast among the people, and by every means in his power he seeks to educate the public mind and the public heart upon the subject he has in hand.

Think how the Pilgrim Fathers of our own New England, steadily, earnestly, and persistently infused their thought into all around them, and thus moulded the character of our most cherished institutions. See how the institutions of New England are moulding the character and destiny of this vast republic,—and ye who can discern the signs of the times, behold how this republic is yet to shape the character and destiny of the world, and give it the glorious light of freedom.

The world is ready to receive light and knowledge and freedom whenever these benefits are presented in sufficiently bright colors to attract its attention, but it never goes after them. The electricity of the universe is not perceived by the multitude except when it flashes on us, accompanied by the reverberating thunder. The besotted inebriate is content to grovel in his swinish condition, with no attempt to free himself from the horrible pit and the miry clay of intemperance, till some friendly hand is stretched out to him and some kind voice whispers in his ear that the fire of manhood is not wholly extinguished. The bond slave mulishly toils for his oppressor without a thought that he can be anything better. He does not seek that knowledge which might make him free. But tell him he is a man and you can not hold him longer. You must take your foot off his neck.

All the light and intelligence and knowledge in the world are preserved and increased and diffused but by the earnest, persistent and energetic efforts of those who possess them, to teach them to others. And Dentistry is no exception to the general rule.

We are aware that there is a class of persons outside the profession who look upon Dentists as a sort of necessary evil that must be endured, and Dentistry as a kind of professional torture to be dreaded more than toothache. They think Dentists a sly, curious sort of geniuses who have got some wonderful secret of their own which they would not divulge for the world, for fear people's teeth would never decay any more, and their craft would be destroyed. They suppose the great object of the profession to be, to produce all the Dental business possible. Consequently when, driven by toothache or deformity, dreaded more, they apply to a dentist at all, they sit down in his chair with fear and trembling, not so much for the operation they wish him to perform as for fear he will perform some operation that will destroy them, that he may have the opportunity to make them some new teeth some other day. They feel no confidence in him. They only employ him because compelled by circumstances, he being the only man who has the instruments and knows the witchery of their use.

Every Dentist knows how disagreeable it is to operate for such patients. He knows they distrust him, and knows too that they distrust him because ignorant of his true interest as well as their own.

Now, why is it that so many persons get such absurd ideas of Dentists and Dentistry? Simply because on no other subject of half so much public worth, is there so much public ignorance as on this. And we regret to be compelled to say it is not confined to the low, or those supposed to be ignorant people, but we find it among all ranks of society, even among scientific men. Yes, even among the medical profession there is only now and then a man who appreciates the subject sufficiently to realize that it is a matter of importance enough to attract the attention of any man of sufficient capacity to fit him for a *doctor*.

Now, who is at fault for all this ignorance and want of appreciation of this branch of surgery? We say the Dentist,—

the Dental profession. We would not be understood to say, that here in the Merrimack Valley we find an ignorant community. Far from it. Scotland's Poet said that "a ploughman is not to be deemed an ignorant man because he knows not how to read, if he knows how to plough." And so we would say that people are not to be considered ignorant because they are not versed in every profession, if they are well informed in their own. Yet we can not help thinking it very desirable as well as useful that the ploughman should possess some intelligent ideas of a great many other things besides ploughing. And one of them should be the nature of his own teeth, their use and abuse. So that when we speak of a want of knowledge on this subject, we do not mean to say, or imply that we practice among an ignorant people; but simply that there is great want of information relative to this branch of science. And why is this so? Why are there so many who understand so little of their true interest, relative to Dentistry and Dentists? Simply because the means of knowledge on this subject have never been presented to them. How can they learn without teachers? And who shall teach them if Dentists will not?

And this brings us back to our starting point, that the Dental profession is responsible for the condition of the teeth of the whole community; and that it is our duty to educate the public in this branch of knowledge.

And, now, my brothers, how shall we meet this responsibility? Shall we go on, content merely to patch up the defective teeth which are presented to us for treatment, and supply artificial substitutes in place of those which are entirely gone? Shall we be content with patch-work and artificial appliances without making an effort to regenerate society on this subject, with the hope that the world may one day present a better Dental aspect than at present?

Every Dentist well knows that a large proportion of the cases presented to him of defective and ruined teeth, are the result of the patients' own carelessness and want of proper attention to those organs. This neglect and mismanagement is chiefly occasioned by the absence of the requisite information.

It is true that there is a vast amount of matter printed on the subject of Dentistry; but most of it is adapted to the reading of Dentists rather than the common reader; and besides, is only published in books and journals, which from their expense, never find their way into the hands of the public; and if they should they would be little better than nothing, being too voluminous—too much lumbered with useless speculations—too much chaff for the wheat. The mass of the people will never have patience to sift it out. They need something by which they can get at the true grain at once,—some concise and definite instructions.

We do not propose to make Dentists of every body because we would have every one understand something of Dentistry, any more than we would have everybody clergymen because all should know something of the bible, religion and theology. But we would simply give everybody such general information on the subject as shall be of practical use in directing them as to what they shall do, and what avoid for the preservation of their teeth.

How much more agreeable it always is for the Dentist to operate for patients who have some intelligent idea of what should be expected of him and his operations, than it is for those who have not: and how perplexing it often is to work for the latter class. They simply understand that a tooth is a tooth and a Dentist is a fool if he cannot preserve one as well as another. They know toothache is toothache and they do not think much of a Dentist who can not apply some medicine that will cure it; no matter from what cause, or what is the condition of the organ. They are aware that their teeth are decaying, but say they will "wait awhile and then have them all done up at once." They do not understand that a tooth filled at the proper time is any better for them than if delayed until but a fragment of it remains.

When they have been to the Dentist and had their teeth filled, and afterwards neglect them entirely, they can not understand why they are not as well preserved as those of some other persons, whom they know, and who are particular to keep their teeth perfectly clean and the fillings bright and pure.

One great object which it seems to us merits the attention of the Dental profession, is to educate the public mind in Dental matters. We do not propose, gentlemen, at the present time, to offer any definite plan for the accomplishment of this object. Our purpose has been simply to call attention to it, hoping that you will give it such thought and action as you may think the importance of the subject demands, rather than that of the feeble and bungling manner in which we have presented it may indicate.

Dr. Allport of Chicago, with the assistance of some other Dentists at the West, contributors to his Journal, is doing something in this direction, by publishing and distributing a little journal on Popular Dentistry, called the "*People's Dental Journal.*"

To us this seems at present one of the best and most efficient means within our reach. We need have no fears that any amount of Dental information possessed by the people will lessen the practice of any well qualified and efficient Dentist while the necessity for his services exists. But the opposite is true, the less intelligence there is, the less attention is given to the teeth, and the less credit the Dentist receives for what he does. It is for our interest as well as for our patients that all the information possible be diffused among the people with whom we practice.

Manchester, N. H., Nov. 3d, 1864.

